# Experimental Study on Plugging Behavior of Degradable Diverters in Partially Open Fracture in Temporary Plugging and Diverting Fracturing

**DOI:** 10.1021/acsomega.3c00689

**Published:** 2023-04-05

**Authors:** Ming Li, Jianchun Guo, Fujian Zhou, Minghui Li, Jiaqi Chen, Hui Liu, Qing Wang, Longqiao Hu

**Affiliations:** † State Key Laboratory of Oil and Gas Reservoir Geology and Exploitation, Southwest Petroleum University, Chengdu 610500, China; ‡ State Key Laboratory of Petroleum Resources and Prospecting, China University of Petroleum at Beijing, Beijing 102249, China; § China United Coalbed Methane Ltd., Beijing 100020, China

## Abstract

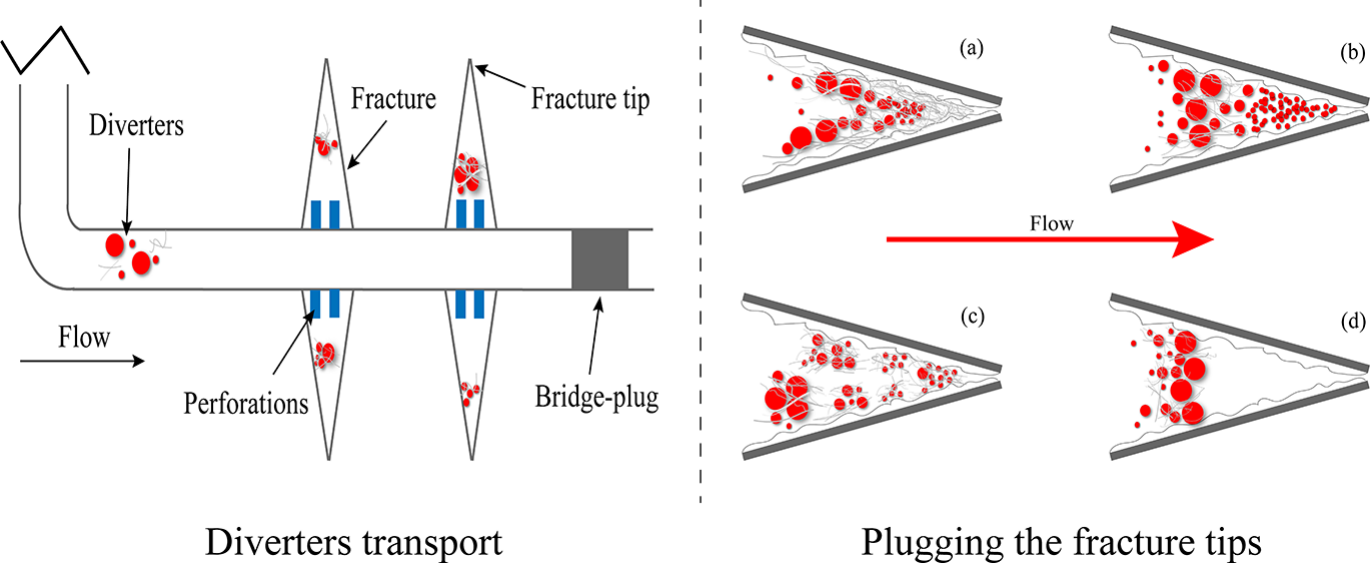

Temporary plugging and diverting fracturing technology (TPDF) has been successfully applied to improve reservoir productivity. In real reservoirs, a considerable number of fractures have relatively rapidly decreasing fracture widths and closed ends. However, the plugging behavior of diverters in this typical fracture called the partially open fracture (POF) is still unclear because of the few related studies. This paper aims to investigate the plugging behavior of diverters at the fracture tip. The 3D-printed fracture model was used to reproduce the partially open fracture, and the morphological characteristics of the partially open fracture and the open fracture were compared based on the scan data. A series of plugging experiments were conducted to monitor the transport behavior of the diverter in partially open fractures through multiple pressure sensors on the fracture model and to investigate the influence of diverter formula and fracture type on plugging behavior. Finally, based on the experimental results, the plugging mechanism of diverters in partially open fractures was analyzed. The plugging experiments show that a higher-pressure distribution appears at the fracture tip when using a combination of fibers and particles, indicating that it is beneficial for the diverter to transport to the tip and form plugging in the fracture, and it should be noted that small changes in particle size and concentration had a significant influence on the plugging performance. Therefore, it is recommended to use a combination of fibers and particles of multiple sizes (maximum particle size not exceeding half of the fracture width) to achieve a better plugging effect. In addition, the plugging behaviors of partially open fractures and open fractures are different. For partially open fractures with widths of 1, 2, and 4 mm, the recommended formula of the diverter is 1 wt % fibers + 1 wt % 0.15 mm particles, 1 wt % fibers + 1 wt % 0.15 mm particles + 1 wt % 1 mm particles, and 1 wt % fibers + 1 wt % 0.15 mm particles + 1 wt % 1 mm particles + 1 wt % 2 mm particles, respectively. The above experimental results provide an experimental and theoretical basis for the application of TPDF in the field.

## 1 Introduction

In its Annual Energy Outlook published in 2021, the U.S. Energy Information Administration mentioned that global oil and gas consumption will increase due to population and economic growth, with global energy consumption increasing by nearly 50% by 2050.^1^ However, they are becoming increasingly difficult and expensive to develop. To secure fuel supplies, operators are beginning to turn their attention to deep tight reservoirs with natural fractures and severe inhomogeneities in the face of declining production from conventional reservoirs.^2−5^ It is well known that hydraulic fracturing is a common production stimulation technology for developing oil and gas resources.^6−9^ However, the results are not satisfactory in reservoirs with poor physical properties.^10−14^ Based on this, some scholars have proposed the temporary plugging and diverting fracturing (TPDF) technology to improve the reservoir production stimulation effect by plugging the well-stimulated zone with diverters and forcing the injected fluid into the poor-stimulated zone to connect the natural fractures and resources.^15−18^ The diverters will be completely degraded and returned to the ground with the formation fluid after the production stimulation, avoiding damage to the reservoir. Compared with other stimulation technologies, TPDF has the advantages of safety, efficiency, and economy and is a more advanced method. TPDF has been successfully applied to the stimulation of Bakken shale reservoirs and Eagle Ford shale reservoirs in North America.^19−23^

Degradable diverters can plug pre-formed fractures and degrade spontaneously after stimulation, and they usually consist of fibers and particles.^24^ Several scholars have investigated the plugging behavior of open fractures through plugging experiments. Abrams^25^ conducted a series of experiments on the matching of pore size and bridging particle size and found that the particle size that can form bridging should be larger than one-third of the pore size. Potapenko et al.^26^ investigated the effect of fracture geometry and diverter formulas consisting of fibers and particles on plugging behavior using a fracture diversion system (FDS). Kefi et al.^27^ investigated the plugging effect of fiber composites using metal cylindrical grooves as an open fracture model. Kang et al.^28^ studied the optimal diverter composition and ratio for fracture plugging with different fracture widths by simulating an open fracture model through a core experimental device. Gomaa et al.^29^ investigated the bridging and plugging ability of particle diverters using disc grooves to simulate fractures and found that the size of particles forming bridging and plugging should be larger than 40% of the fracture width. Other studies^11,13,30−32^ performed a series of plugging experiments with acid-etched fracture models to investigate the plugging behavior of fiber and particle formulas on different fracture surfaces. Yang et al.^33^ conducted plugging experiments using a visualization experimental system to summarize the plugging behavior of fibers and particles in flat fractures in four stages. Zhao et al.^34^ comprehensively evaluated a variety of plugging experiments and summarized the plugging mechanism of material diverters.

In the field of reservoir engineering, the study of diverter behavior in partially open fractures has received limited attention. Previous studies have primarily focused on the plugging behavior of diverters in open fractures without fracture tips. However, many fractures in real reservoirs are partially open, meaning they have tips at their ends, which can be caused by overburden pressure or other reasons.^35−37^ Therefore, the current study can only explain the plugging behavior of the diverter in the open fracture (which did not reach the tip of the fracture). Few pieces of literature have studied the plugging mechanism of partial open fractures. The existing studies that we can find were limited to gel plugging behavior.^38,39^ Further investigation is needed to understand the diverter plugging behavior when it is injected into the tip of a partially open fracture, as it is likely to differ significantly from its behavior in an open fracture.

The current study aims to investigate the plugging mechanism of diverters in partially open fractures with fracture tips in tight sandstone reservoirs. The partially open fractures were reproduced through 3D printing technology, and their morphological characteristics were described based on scanned surface profile data. A series of plugging experiments were conducted to analyze the transport behavior of the diverter at the fracture tip and its plugging performance under various diverter formulas and different fracture types. The results of the experiments were used to summarize and explain the plugging mechanism of diverters at the fracture tip. This study provides valuable insights into the conformance control of tight sandstone reservoirs with partially open fractures.

## 2 Methodology

**Figure 1:**
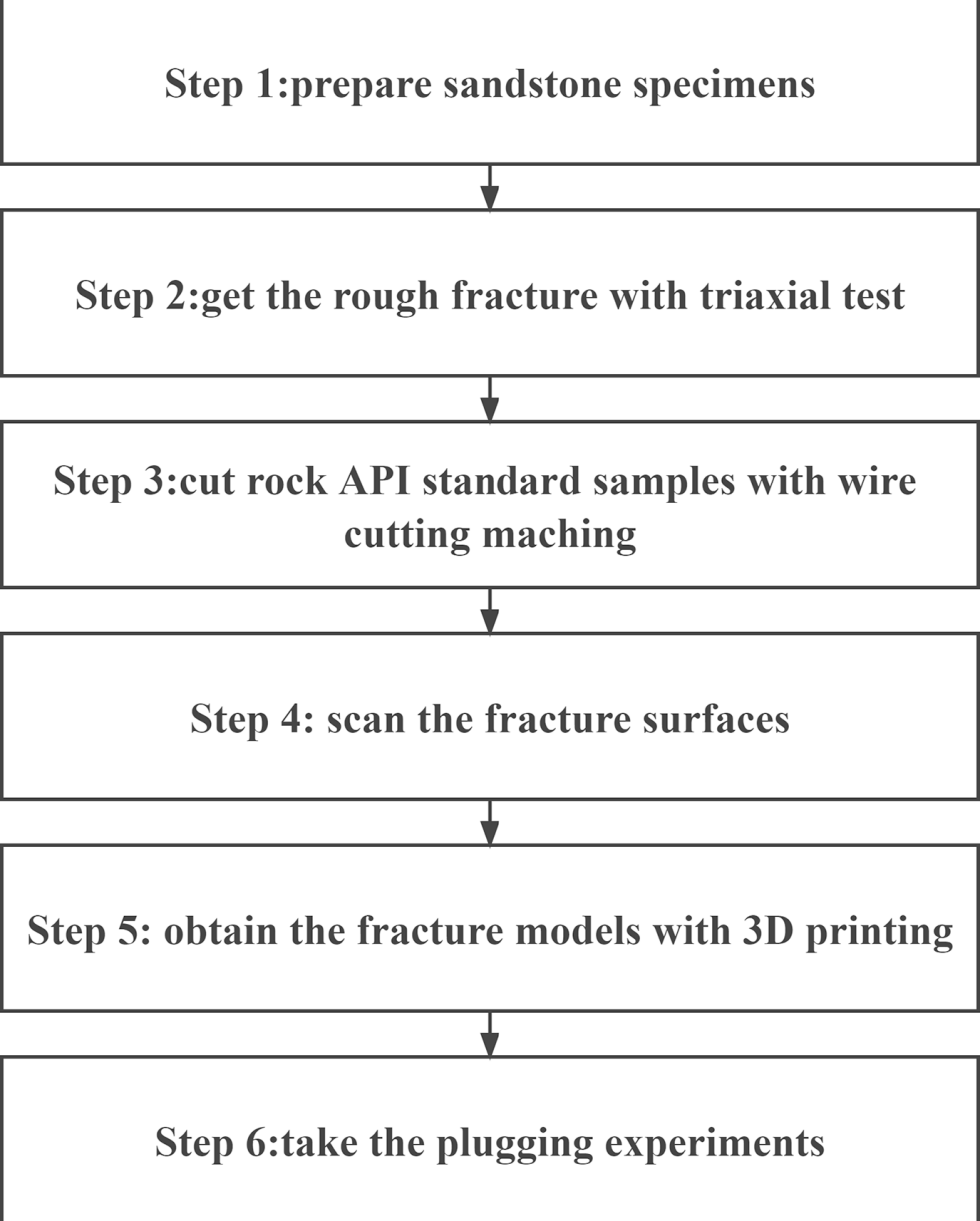
Step of experimental procedures.

**Figure 2:**
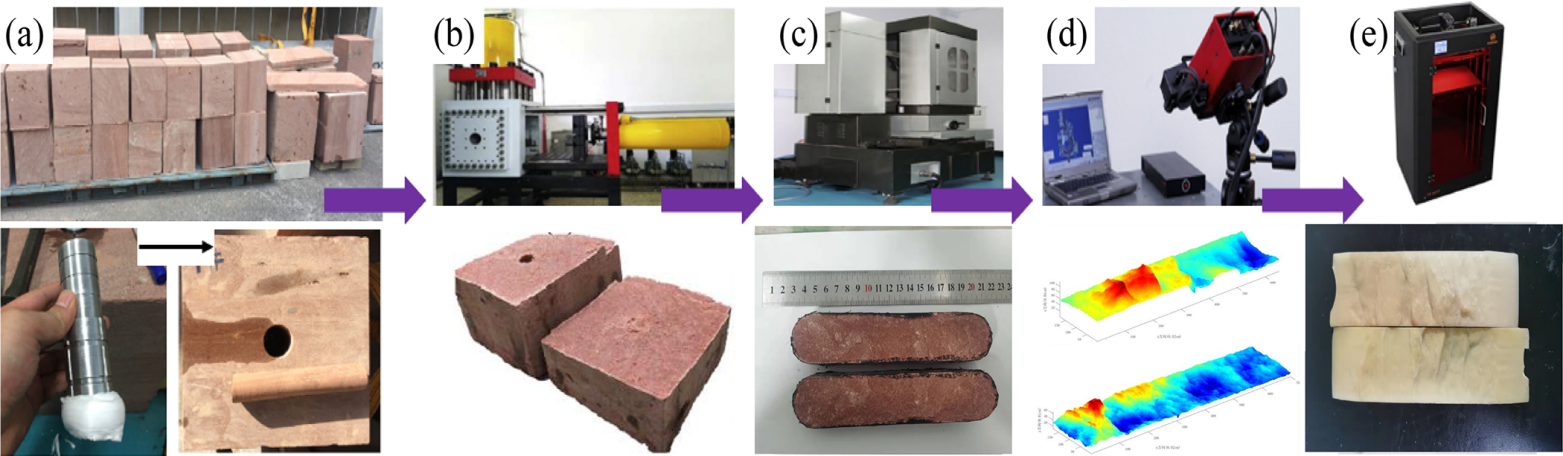
The flow diagram of experimental apparatus for 3D printing models. (a) Sandstone specimen from Tarim oilfield; (b) true tri-axial fracturing system; (c) wire-cutting system; (d) micro-nano laser scanning system; (e) 3D printing system.

In order to investigate the characteristics of partially open fractures, the three-dimensional digital data of fracture surface morphology were analyzed deeply and a series of experimental studies on the temporary plugging behavior of degradable diverters were conducted based on the reproduced partially open fracture model. Figure 1 lists the entire experimental step,^30^ and Figure 2 shows the preparation process and application steps.^13,16,31^ First, nearly uniform sandstone samples were collected from the Kuqa foreland basin of the Tarim oil field in western China and cut into 300 mm × 300 mm × 300 mm blocks by a wire cutting system. A central hole is drilled in the center of the cube, and a steel pipe is placed to simulate the wellbore. Second, real hydraulic fracturing fractures are produced by a triaxial fracturing system. Third, the fractured rocks used in triaxial fracturing experiments are cut into API standard specimens by a wire cutting system. Fourth, the rock samples after wire cutting are scanned by the micro-nano laser scanning system, and highly accurate data on fracture morphology are obtained. Then, through the 3D printing system, the 3D printing model is obtained. Finally, based on the fracture model and plugging evaluation system, a series of experiments are conducted to study the plugging law of partially open fractures.

### 2.1 Experimental Materials

#### 2.1.1 The 3D Printing Fracture Model

**Figure 3:**
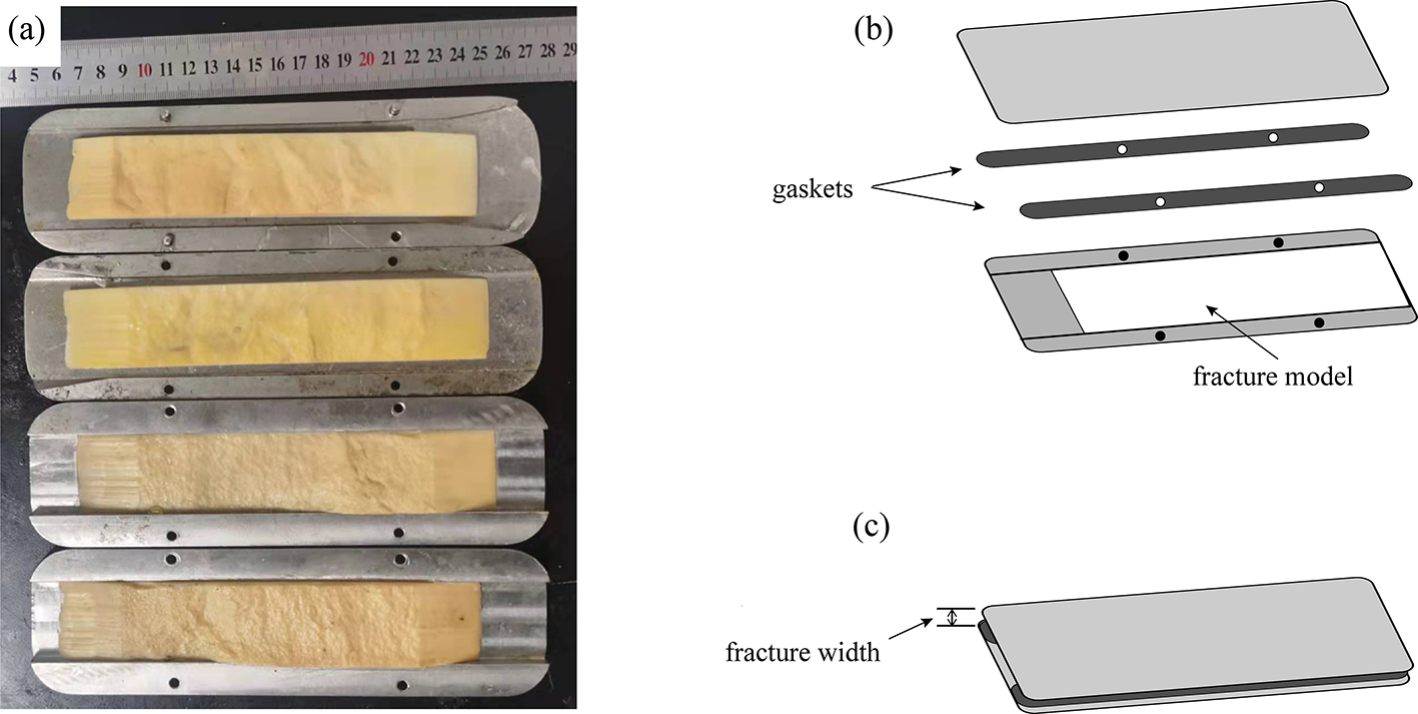
3D-printed fracture model. (a) Fracture models; (b) side view; (c) fracture models after installation.

Due to the influence of high-pressure difference and fluid, the real samples in the plugging experiment may be damaged, so the 3D-printed fracture model can ensure the repeatability of the experiment. After the fracturing experiment, the two surface morphologies of the fractures are scanned by a 3D scanner and the accuracy of the scanner can reach 0.0006 mm. On this basis, two model plates with actual surface roughness are printed by a 3D printer. The material of each board is photosensitive resin with enough resistance to deformation, and the size is 180 mm × 45 mm. The actual picture of the fracture model is shown in Figure 3. Each steel plate is then loaded into a metal container to simulate the fracture space. The fracture entrance is wedge-shaped to avoid excessive accumulation of the diverters at the entrance and facilitate the diverters to enter the fracture. The width between the upper and lower model plates is controlled in the 0.05–5 mm range by increasing the thickness of the gasket.

#### 2.1.2 Carrying Fluid

In the plugging experiment, hydroxypropyl guar gum fracturing fluid was selected as the carrying fluid to carry the diverters. Based on field application, the formula of the carrying liquid consists of 0.4 wt % hydroxypropyl guar gum, 0.03 wt % citric acid, 0.5 wt % discharge surfactant, 0.15 wt % cross-linking agent, 0.2 wt % cross-linking regulator, and other trace additives.^11,16^ The viscosity of carrying fluid measured by rotary viscometer is approximately 200 mPa·s.

#### 2.1.3 Degradable Diverters

**Figure 4:**
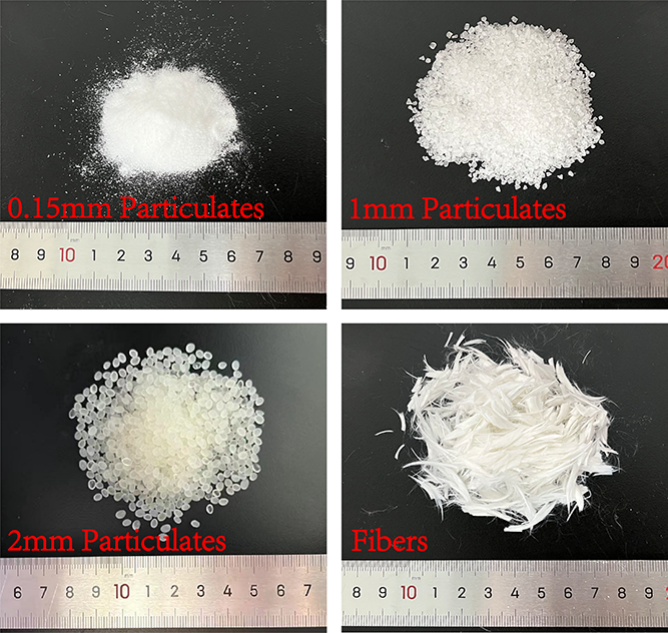
Four types of diverters in the experiments.

The degradable diverters used in the field can be divided into fibers and particles in terms of shape.^30^ As shown in Figure 4, fibers and particles were used as degradable diverters in this experiment. Fibers and particles are made of copolymers of lactic acid and glycolic acid, which can be degraded automatically at the formation temperature but are insoluble in water at room temperature. Particularly, the fiber length is 6 mm, the diameter is 10 μm, and the particle diameters are 0.15 mm (100 mesh), 1 mm, and 2 mm, respectively.

### 2.2 Plugging Experimental System and Procedure

**Figure 5:**
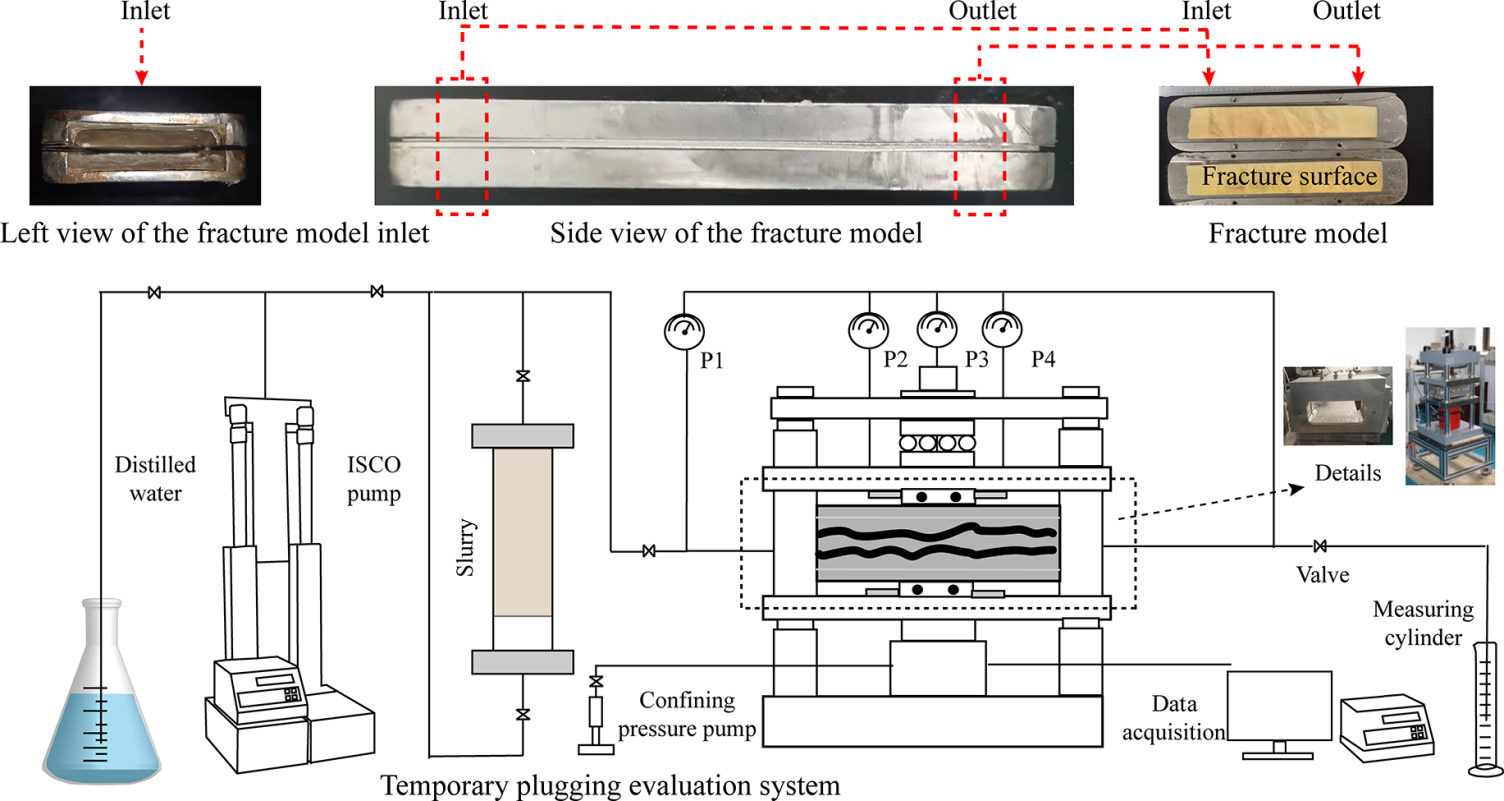
Schematic of the temporary plugging experiments.

As shown in Figure 5, the plugging experimental system consists of a fracture conductivity cell (including a fracture model), an ISCO pump, a confining pressure pump, four pressure gauges, and a data acquisition system. The mixture of diverters and carrying liquid is injected into the fracture model through the high-pressure pipeline using the ISCO pump. The maximum injection rate of the ISCO pump is 102 mL/min, and the flow accuracy is 0.06%. Four electronic pressure gauges are installed in the fracture model to monitor the pressure at different positions in the plugging experiment. The balance is used to measure the quality of the effluent. The sampling interval of the whole experiment is 2 s. In particular, the inner diameter of the pipeline was increased to 13 mm in the experiment to prevent the diverters from blocking the pipeline. The experimental procedure is as follows:

(1) Prepare the carrying fluid with diverters according to the experimental scheme, and then inject it into the intermediate container and connect the pipeline;
(2) Install a steel plate gasket on both sides of the 3D printing fracture plate, set the fracture width, and then put it into the conductivity cell.
(3) Add the confining pressure of 15 MPa to the conductivity cell by the confining pressure pump, start the ISCO pump to inject the carrying fluid into the conductivity cell at a constant injection rate, and record the pressure value of the gauges.
(4) Stop the experiment and record the data when the pressure reaches 10 MPa or the injection volume reaches the design value.

### 2.3 Experimental Schemes

In this paper, 12 groups of experiments are conducted based on the fracture model and plugging evaluation system shown in Figure 5. All the experiments are divided into two types: open fracture model and partially open fracture model. 1, 2, and 4 mm are selected as typical fracture widths. Three kinds of diverters, fiber, powder (100 mesh particles with 0.15 mm particle size), and particles, were also used. The concentration of each diverter was set at 0 or 1.0 wt %. Among them, group nos. 1–2 investigated the transport behavior of the diverters. Group nos. 1–9 investigated the effect of different diverter concentrations on the plugging effect. Group nos. 10–12 were conducted as a parallel experiment to study the plugging law under different fracture types.

## 3 Results and Discussion

In this part, the surface morphologies of partially open fractures and open fractures are compared, the transport behavior of diverters is analyzed, and the effects of diverter concentration and fracture morphology on the plugging effect are investigated. To be consistent with previous studies, an inlet pressure of 10 MPa is taken as the basis for forming an effective plugging inside the fracture. Finally, the plugging mechanism of diverters in partially open fractures is proposed.

### 3.1 Features of Partially Open Fracture

**Figure 6:**
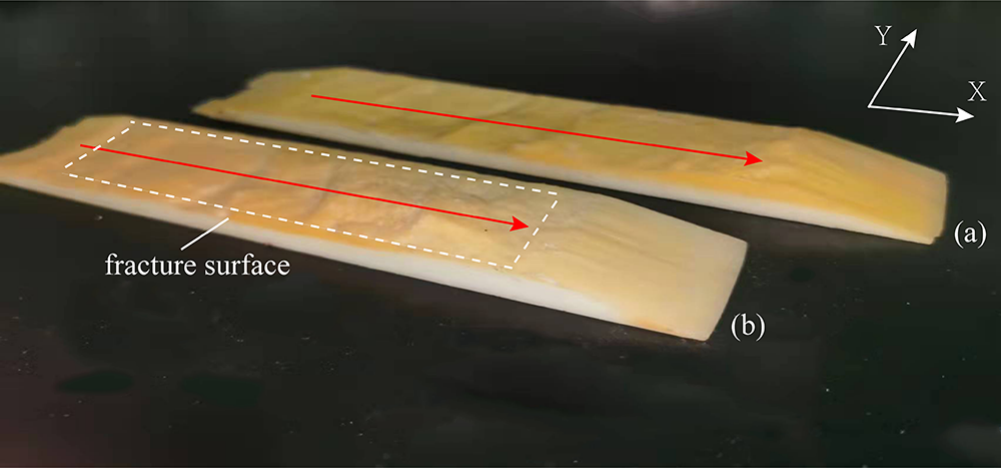
3D-scanned surface morphologies. (a) Open fracture; (b) partially open fracture.

The scanned surface morphologies of open fractures and partially open fractures are compared as shown in Figure 6, where Figure 6a shows the surface morphology of the open fracture and Figure 6b shows the surface morphology of the partially open fracture. It can be seen that there are many uneven surfaces and sharp protrusions on the open fracture surface, indicating that the fracture is brittle. The surface morphology of the partially open fracture is similar to that of the open fracture, but the outlet width is significantly smaller than the inlet width because the original parallel flow channel decreases rapidly due to the sharp decrease of the fracture width.

**Figure 7:**
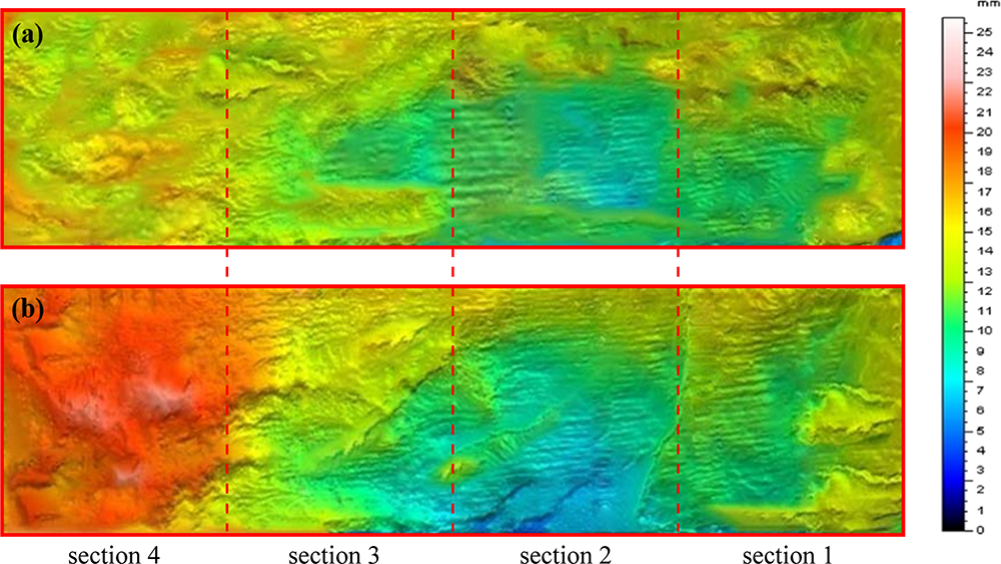
3D fracture surface. (a) Open fracture; (b) partially open fracture.

Figure 7 shows the magnified details of the fracture surface morphology. Along the flow direction, there are a large number of pits and flow channels on the surface of open fractures and partially open fractures. In order to facilitate the subsequent study, the whole fracture model is artificially divided into four sections and the pressure changes of each section are recorded in the course of the experiment.

**Figure 8:**
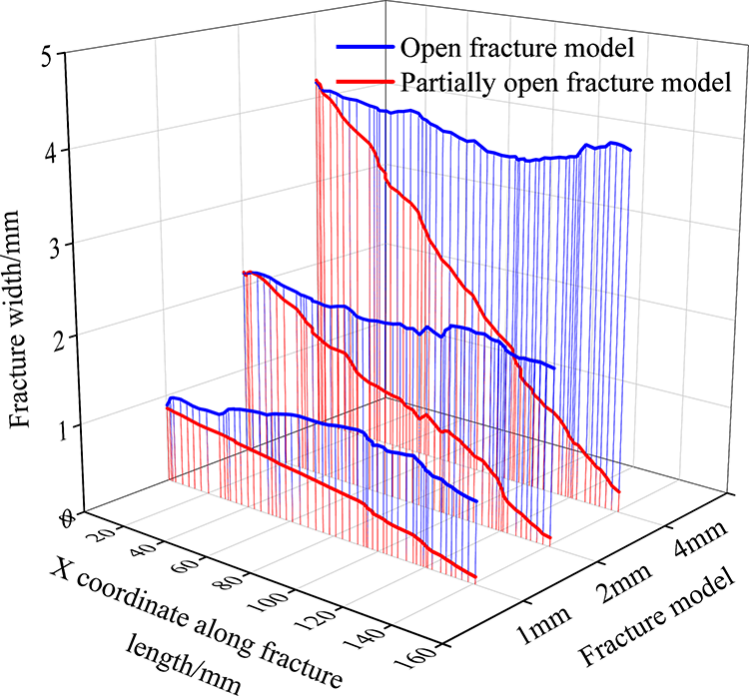
Surface profiles along the *X*-direction, at *Y* = 23 mm.

To further analyze the morphological characteristics of the fracture surface, a fracture parallel to the flow direction (*X*-direction) was extracted from the fracture morphology scan results as an independent cross section. The profile of *Y* = 23 mm is drawn in Figure 8 to observe the variation of the complete fracture morphology. It can be seen from the figure that along the flow direction (*X*-direction), the fracture surface is rough with zigzag craters and peaks. As the fracture length increases, the width of the open fracture stays near the initial fracture width, while the width of partially open fractures decreases sharply and almost closes at the end of the fracture, leaving only a narrow fracture width, which increases the flow resistance. In addition, it can be seen from the figure that the morphology of different parts of the open fracture has craters and convex peaks, and the morphological changes are similar without much difference, so the errors caused by the experimental material can be eliminated in the subsequent experiments.

### 3.2 Transport Behavior of the Diverter

**Figure 9:**
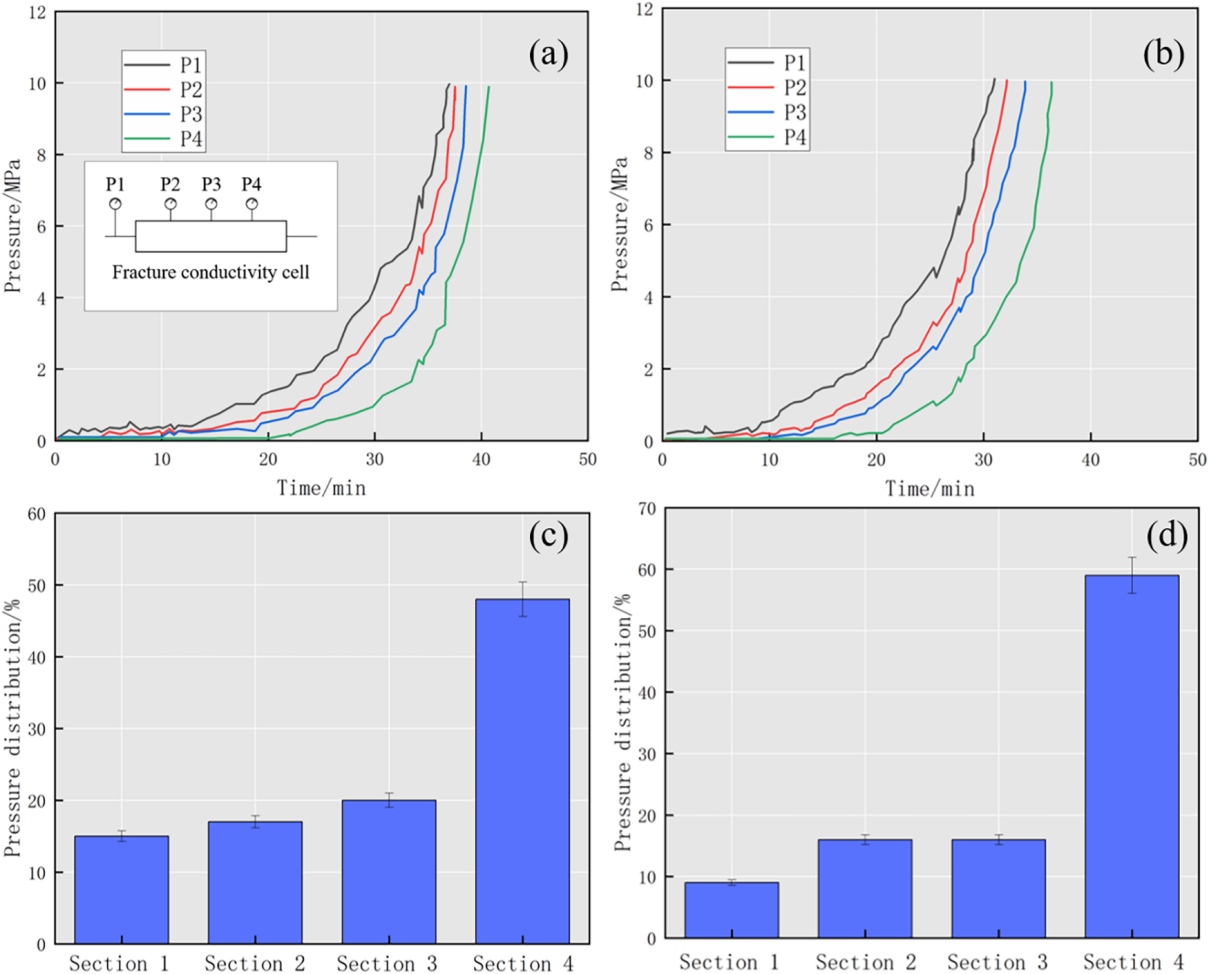
Pressure curve and pressure distribution of each section. (a) Pressure curve of group no. 1; (b) pressure curve of group no. 2; (c) pressure distribution of group no. 1; (d) pressure distribution of group no. 2.

The transport dynamic of diverters in partially open fractures was analyzed by pressure response. Two groups of experiments (nos. 1–2) were conducted using the parameters listed in Table 1. The diverter was injected into the fractures at the injection rate designed for the experiments, and the change of pressure at different sections was recorded. The beginning of the pressure rise indicated that the diverter started to form plugging at the detection point of the pressure gauge. Figure 9a shows the pressure variation in different sections of the fracture model in group no. 1. The fracture width in this experiment is 1 mm, and the diverter formula is 1 wt % F. When the injection started, the inlet pressure (P1) started to fluctuate and did not increase significantly, indicating that the diverter entered the fracture and transported to the first section but did not form an effective plugging, while the pressure in the other three sections remained zero. After 4 min of injection, P2 began to fluctuate and the trend was the same as in P1. It indicates that the diverter transported along the fracture to the second pressure measuring point (P2) but did not form an effective plugging. When the injection time reached 10 and 20 min, respectively, the front of the diverter reached the third and fourth pressure measuring points and P3 and P4 started to fluctuate. It is worth noting that after 10 min, the pressure at all four sections increased significantly and finally reached 10 MPa at 40 min, indicating that the effective plugging of the diverter was formed in sections 3–4. The slope of the four pressure curves can be compared as follows: P4 > P3 > P2 > P1, which indicates that the pressure increment mainly occurs in the fourth section and the pressure increment in the first part is the smallest. The calculated pressure distribution diagram also confirms this point of view. The pressure distributions of sections 1–4 are 15, 17, 20, and 48%, respectively. The analysis shows that the loose plugging zone formed in sections 1–2 makes a small contribution to the plugging pressure while the tight plugging zone formed in sections 3–4 bears the main plugging pressure.

**Table 1:** Parameters of Plugging Experiments

classification	experiment group no.	fracture width (mm)	injection rate (mL/min)	fiber concentration	0.15 mm particle concentration	1 mm particle concentration	2 mm particle concentration
partially open fracture model	1	1	40	1			
	2	1	40	1	1		
	3	2	40	1	1		
	4	2	40	1		1	
	5	2	40	1	1	1	
	6	4	40	1	1		
	7	4	40	1	1	1	
	8	4	40	1	1		1
	9	4	40	1	1	1	1
open fracture model	10	1	40	1	1		
	11	2	40	1	1	1	
	12	4	40	1	1	1	1

In group no. 2, the fracture width is 1 mm and the diverter formula is 1 wt % F + 1 wt % 0.15 mm P. The pressure curve and pressure distribution are shown in Figure 9b. In this experiment, the injection time corresponding to the response of P2, P2, and P4 was 4, 8, and 16 min, respectively, and the transport rate of diverters was faster than that of group no. 1, indicating that compared with a single fiber, the combination of particles and fibers facilitate the transport of diverter in the fracture. In total, the diverter of 35 min was injected and the pressure at all four sections reached 10 MPa. The pressure distribution diagram shows that the pressure distribution of sections 1–4 is 9, 16, 16, and 59%, respectively. Compared with the data from group no. 1, the first three parts show a lower contribution to the plugging pressure during the injection process, and the final section bears the majority of the plugging pressure. Due to the combined formula of particles and fiber, the diverter is more likely to transport and form a plugging at the fracture tip.

### 3.3 The Effect of Diverter Formula

**Figure 10:**
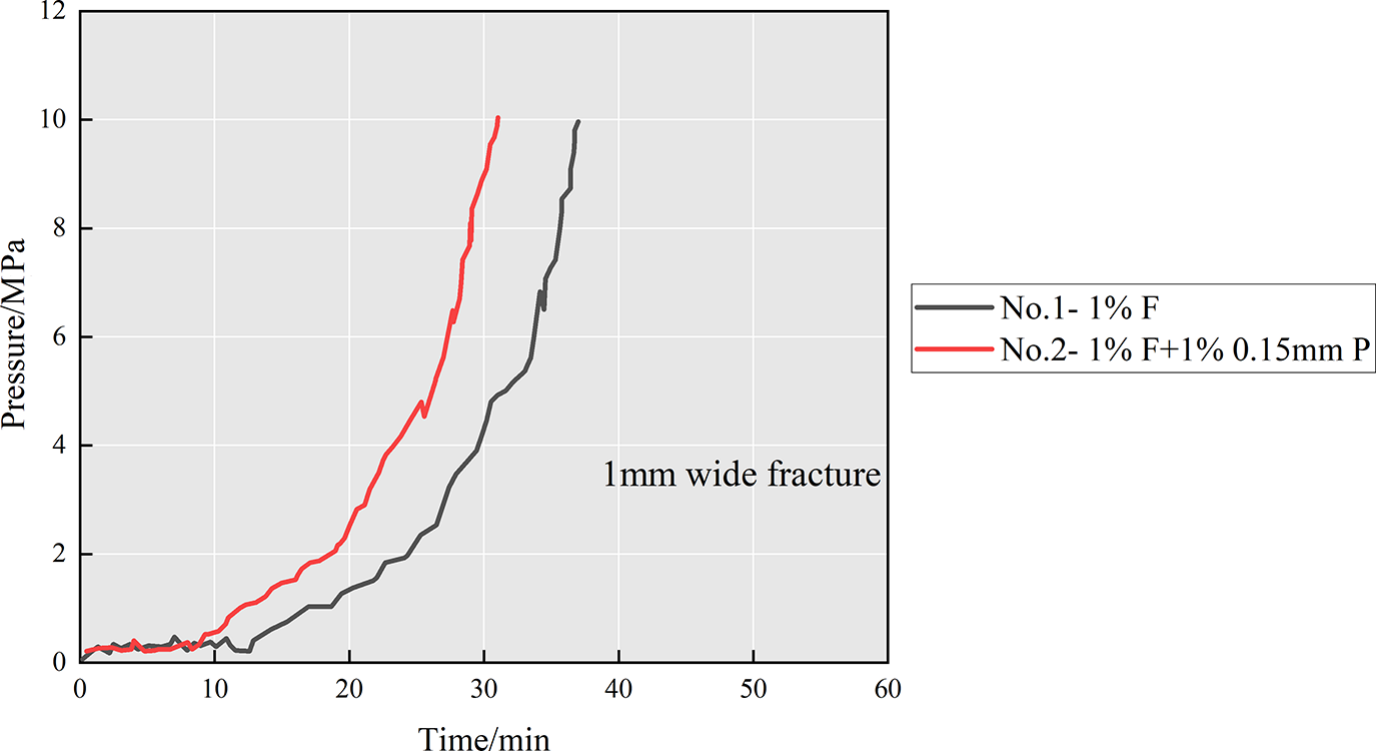
The inlet pressure curve for group nos. 1–2.

The formula of diverters has an important influence on the plugging effect. To evaluate the plugging performance of different formulas of diverters, nine groups of plugging experiments with different formulas and fracture widths were conducted, in which the fracture widths of group nos. 1–2 were 1 mm, those of group nos. 3–5 were 2 mm, and those of group nos. 6–9 were 4 mm. During the experiments, the injection rate was 40 mL/min and the inlet pressure (P1) was recorded as the plugging pressure. From Figure 10, it can be seen that both the fibers and the combination of fibers and particles can effectively plug 1 mm fractures. For the diverter formula with 1 wt % F, the inlet pressure fluctuated slightly with almost no increase in the first 12 min and then increased approximately exponentially with time to 10 MPa, indicating that the fiber gathered at the fracture tip to form an effective plugging zone. When the formula of diverters was 1 wt % F + 1 wt % 0.15 mm P, the plugging effect was further improved and the inlet pressure increased rapidly from 8 min to 10 MPa, indicating that the composite diverter (fibers and particles) can plug the fracture better. This can be attributed to the synergistic effect of fibers and particles, where the particles first form a bridging plug in the narrow part of the fracture, when the plugging zone can withstand a certain pressure, and then the fibers further attach to the bridging gap to form a tight plugging zone. Compared with group no. 1, group no. 2 formed effective plugging faster and the efficiency was increased by 19%. Considering the plugging formation time and plugging pressure, the optimal plugging formula for a partially open fracture of 1 mm is 1 wt % F + 1 wt % 0.15 mm P.

**Figure 11:**
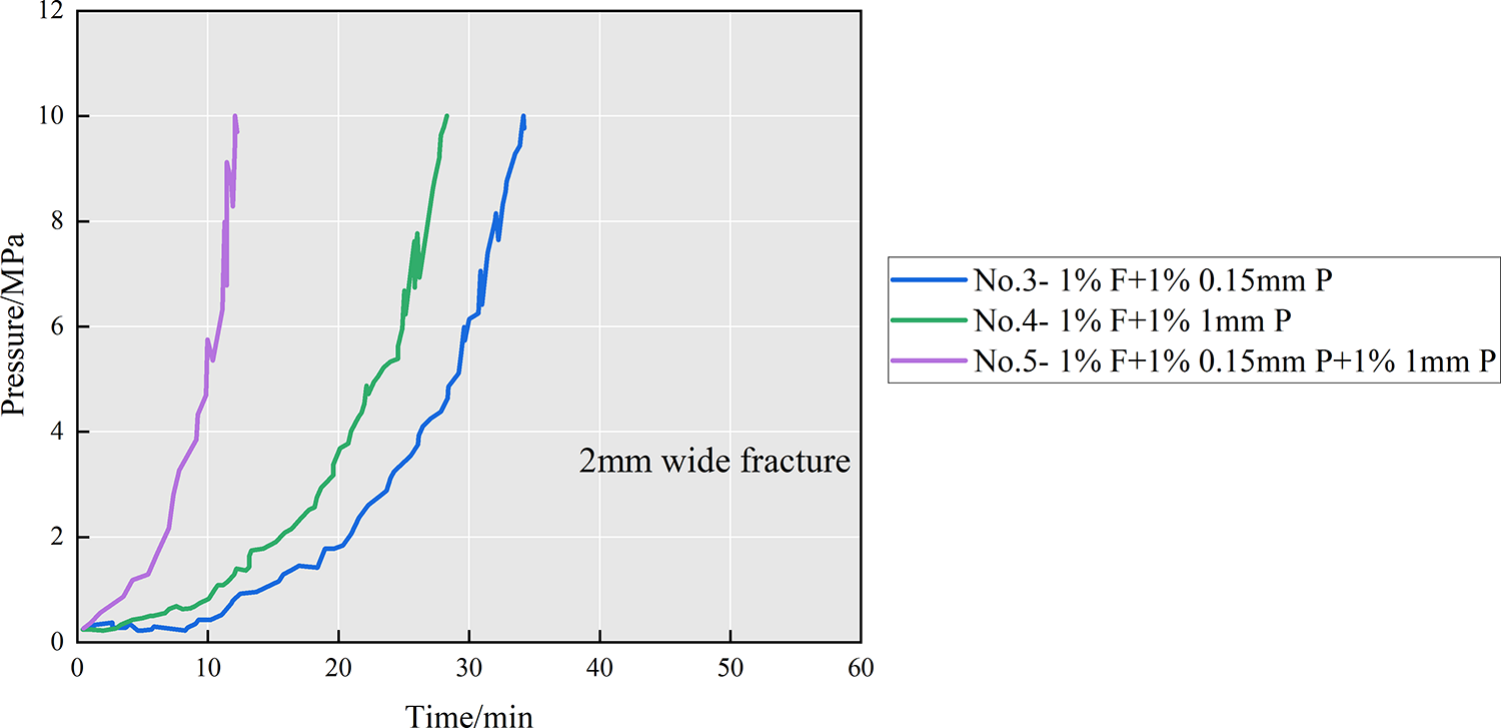
The inlet pressure curve for group nos. 3–5.

Experiments on the plugging effectiveness of the combinations of fibers and two kinds of particles were conducted in 2 mm fracture width experiments to further investigate the plugging process. The same fiber concentration formula was used for all experiments, but the difference was the change in the type and concentration of the particles. The inlet pressure variation is observed in Figure 11. When using the diverter formula with 1 wt % F + 1 wt % 0.15 mm P, the inlet pressure remained essentially constant for the first 9 min and reached the upper limit of 10 MPa at about 34 min. When the particle size was increased and 1 wt % F + 1 wt % 1 mm P were used, the inlet pressure increased significantly after 3 min and reached 10 MPa at about 28 min. This indicates that increasing the diverter particle size helps to start the pressure of plugging and accelerate the formation of the plugging. Continuing to improve the diverter formula, the inlet pressure in group no. 12 increased rapidly after 1 min and reached 10 MPa more than 15 min faster than the single-particle size formula when the fiber and two-particle size formulas were used.

By comparing the experimental pressure rise of each group, it is found that the plugging contribution of 0.15 mm particles to the 2 mm fracture is limited. With 1 mm particles, a bridge plugging zone is more likely to form during the injection process, which accelerates the inlet pressure change and leads to a mild pressure rise. The fastest pressure rise trend in group no. 5 is mainly due to the reason that larger particles tend to bridge at narrow fractures during injection, forming a loose plugging zone. Smaller particles and fibers can form effective plugging by filling the gap, thus accelerating the growth of the plugging zone. To sum up, for a partially open fracture with a width of 2 mm, the optimal formula is 1% F + 1% 0.15 mm P + 1%1 mm P.

**Figure 12:**
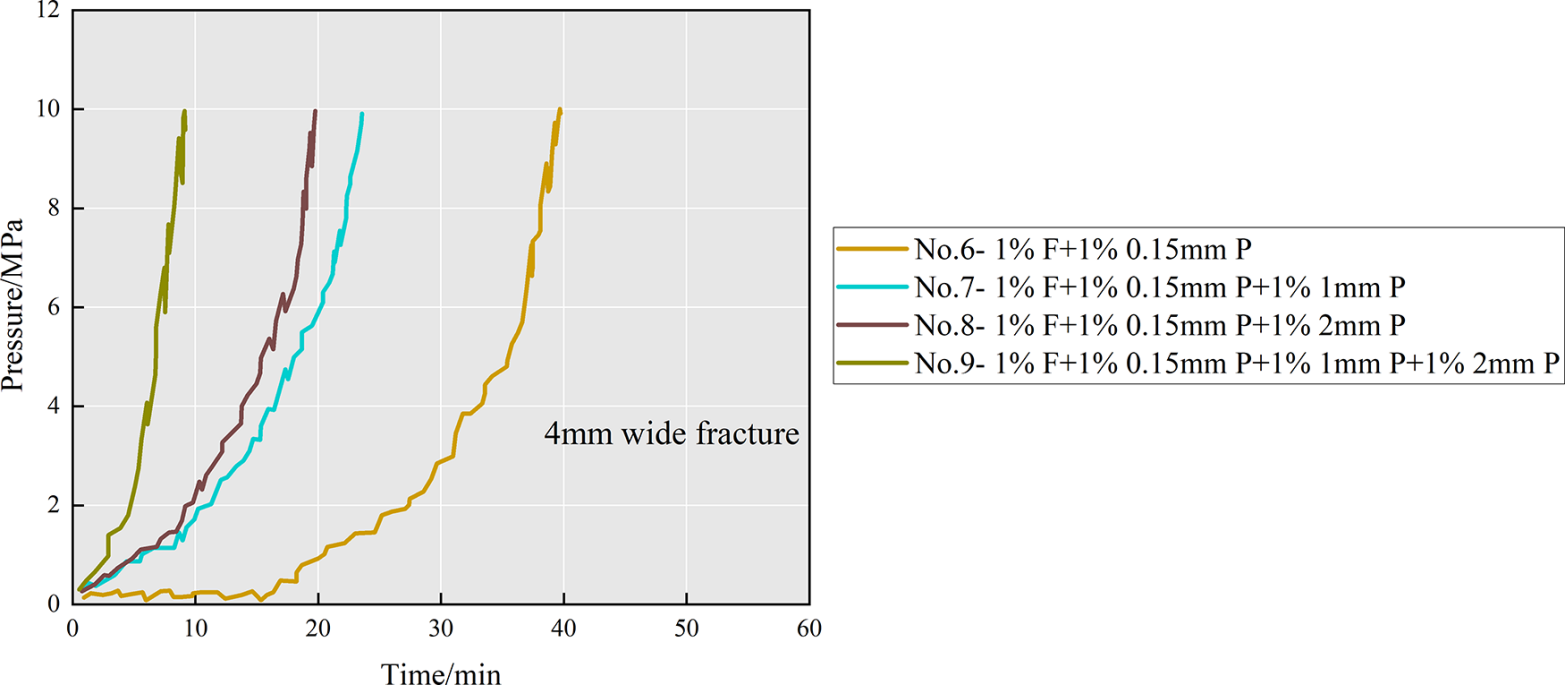
The inlet pressure curve for group nos. 6–9.

It is well known that plugging becomes more difficult as the fracture width increases.^13^ However, the effect of the ratio of fibers to composite particles on the plugging effect at a certain fracture width is not clear. Therefore, group no. 6 (1% F + 1% 0.15 mm P), group no. 7 (1% F + 1% 0.15 mm P + 1% 1 mm P), group no. 8 (1% F + 1% 0.15 mm P + 1% 2 mm P), and group no. 9 (1% F + 1% 0.15 mm P + 1% 1 mm P + 1% 2 mm P) were conducted at a fracture width of 4 mm. The inlet pressure during the experiments is shown in Figure 12. Compared with group no. 6, the pressure in group no. 9 increased rapidly in the early and late stages, and the final plugging time was about 9 min. The results show that the larger the number of large particles added, the earlier the plugging formation time and the addition of composite particles accelerates the plugging formation in the late stage. This is due to the bridging of large and medium particles in the narrow region of the front fracture, followed by the flow of fibers and small particles to fill the gap in the bridge. To sum up, considering the better plugging effect, for a certain fracture width, it is recommended that the ratio of fibers, small particles (0.15 mm particles), medium particles, and large particles (particle diameter is half of the fracture width) is 1:1:1:1.

### 3.4 The Effect of Fracture Type

**Figure 13:**
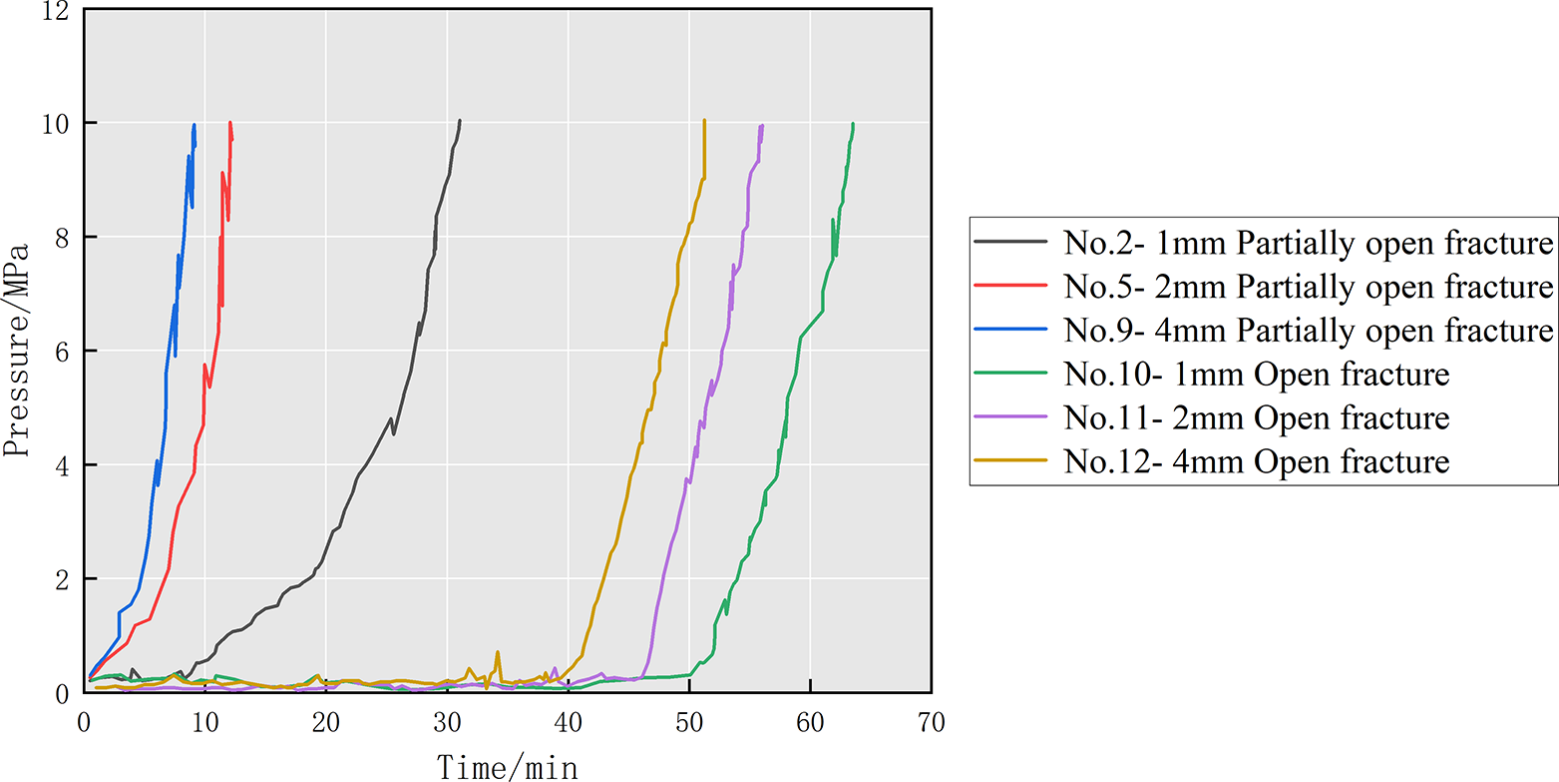
The inlet pressure curve for experimental control group nos. 10–12.

From the above experimental results, it can be known that the surface morphology of the partially open fracture model will significantly affect the transport and plugging of the diverter in the fracture. In this study, to further reveal the effect of fracture types on the plugging behavior, open fractures were used as the experimental control groups (nos. 10–12). Compared with the partially open fracture experimental groups, only the surface morphology was different and other experimental conditions were the same. Figure 13 shows the inlet pressure curve for the control experiment. Compared with the rapidly increasing trend of the pressure in the partially open fracture, the pressure in the open fracture all experienced fluctuations for more than 40 min with almost no significant increase and then rapidly rose to 10 MPa, indicating that there is no effective plugging zone formed in the fracture in the early stage. Analysis of the three groups of control experiments shows that the diverter formulation that can effectively plug partially open fractures does not easily form a plugging zone in the open fractures. The main reason is that the minimum fracture width (FW_m_) of open fractures does not tend to decrease continuously but maintains a high level,^11,40^ and a larger FW_m_ is not conducive to the bridging of particles and the bonding of fibers. Fortunately, a sufficient diverter was injected into the open fracture and an effective plugging zone eventually formed in the fracture. By counting the diverter consumption after the experiment, it is found that the open fractures have the highest diverter consumption, which is almost three times higher than that of the partially open fractures.

**Figure 14:**
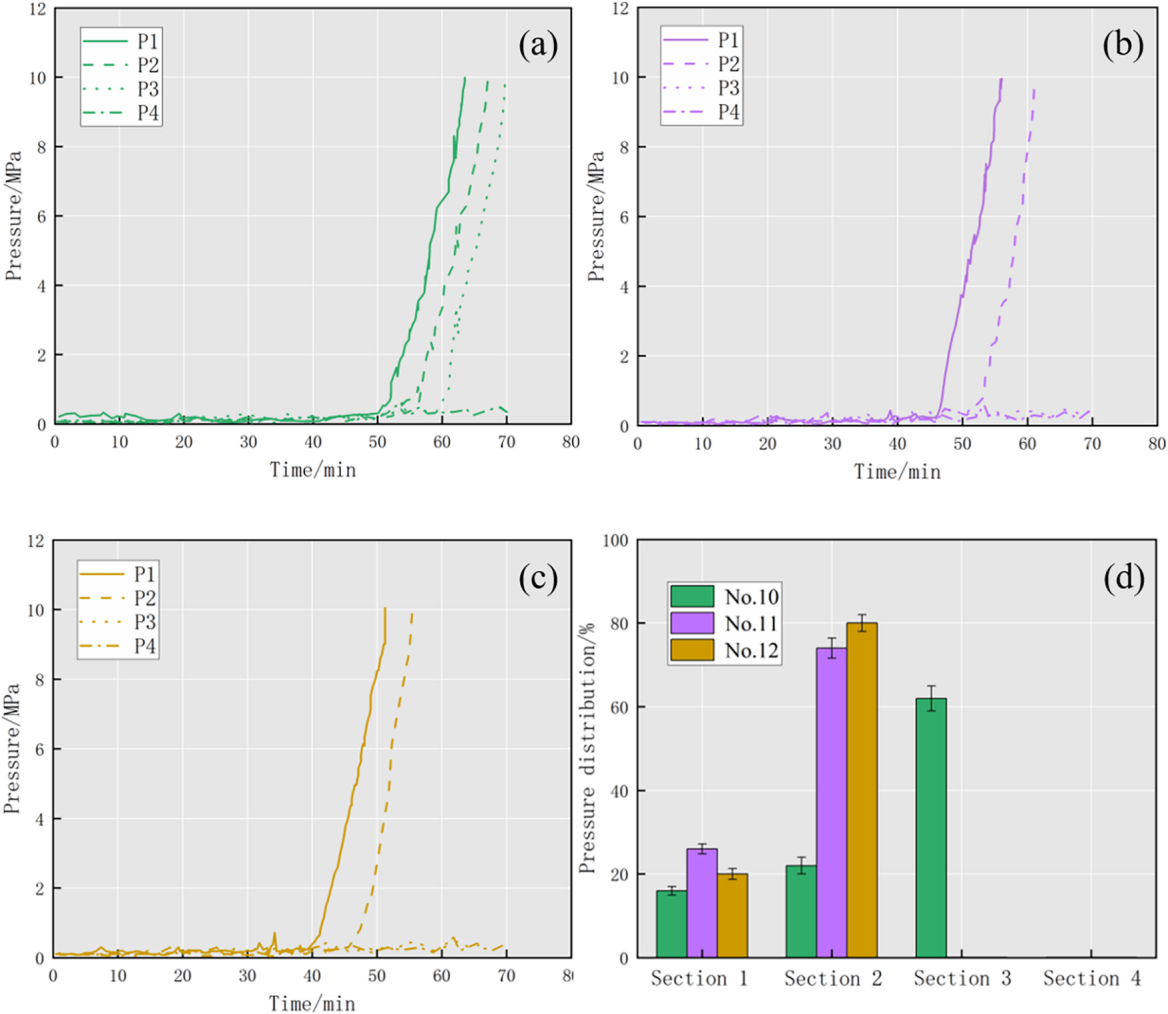
Pressure curve and pressure distribution of each section. (a) Pressure curve of group no. 10; (b) pressure curve of group no. 11; (c) pressure curve of group no. 12; (d) pressure distribution of group nos. 10–12.

To further clarify the plugging process of the diverter in the fracture, the data changes of four pressure gauges during the open fracture plugging process were recorded, as shown in Figure 14. The plugging formula of the 1 mm fracture used a combination of fiber and one particle size (Figure 14a), and the pressure of the first three sections (P1–P3) increased rapidly to 10 MPa after a long period of gentle fluctuation during the injection process, indicating that the first three sections are plugged successfully. The fourth section of the pressure was kept at a low level from the beginning to the end and did not form an effective plugging. The pressure distribution also confirms this conclusion, the pressure of the four sections is 16, 22, 62, and 0% respectively, and the third section bears most of the pressure, indicating that the plugging zone is mainly formed in the third section. The plugging formula for a 2 mm fracture was the combination of fiber and two kinds of particle sizes (Figure 14b), and the plugging effect was better than that of a 1 mm fracture. The difference is that only the first and second sections of the pressure (P1, P2) finally reached 10 MPa while the pressure in the third and fourth sections never increased. In terms of pressure distribution, the pressure of the four sections is 26, 74, 0, and 0% respectively, indicating that the plugging zone is mainly formed in the second section. The analysis shows that due to the addition of two-particle sizes, the fracture surface, which originally cannot be bridged by a single particle, is affected by the transport and collision of two particles, which increases the possibility of bridging with diverters, so the plugging position is changed to make the plugging happen earlier. On this basis, the plugging formula of the 4 mm fracture added three particle sizes (Figure 14c) and the pressure change trend was similar to that of the 2 mm fracture. While the pressure in the first two sections (P1, P2) reached 10 MPa, the pressure in the last two sections did not increase. The pressure distribution of each part was 20, 80, 0, and 0%, and the plugging zone was mainly formed in the second section. The plugging trend of the 4 mm fracture was similar to the 2 mm fracture plugging, but the pressure initiation and plugging time were accelerated. The analysis shows that the combination of using multiple particle sizes increases the intensity of bridging the plugging and contributes to forming the plugging rapidly. According to the above results, there are some differences in the plugging behavior of partially open fractures and open fractures, and the diverter formula for plugging partially open fractures may not be applicable for an open fracture. By finding a suitable diverter formula, the plugging effect of fracture can be greatly improved.

### 3.5 Temporary Plugging Mechanisms of Diverters in Partially Open Fractures

**Figure 15:**
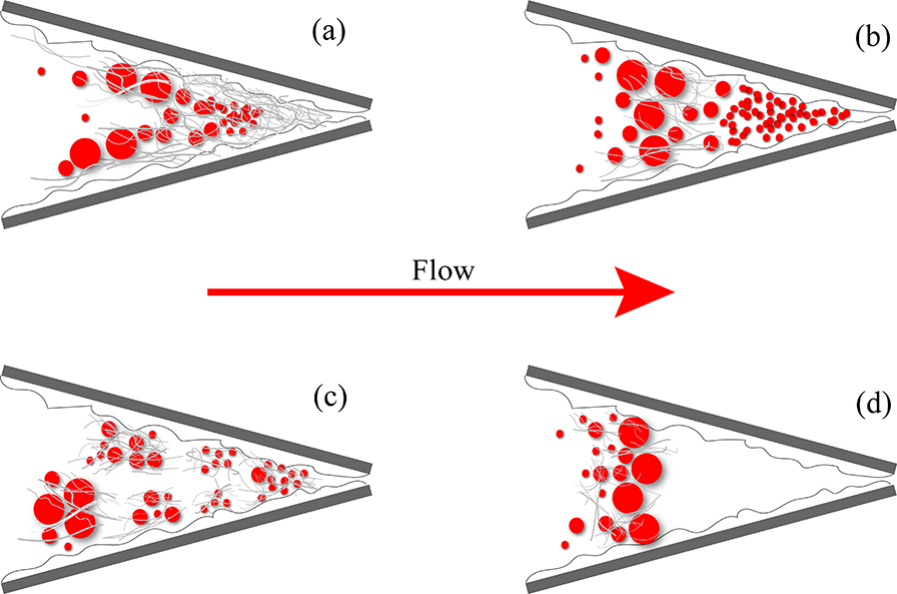
Plugging mechanism of diverters in partially open fractures. (a) Fibers attached to the surface and filled the fracture tip; (b) small particles filled the fracture tip; (c) a mixture of fibers and particles filled the fracture tip; (d) the fracture tip was not filled.

Through the above experiments and discussion, the plugging mechanism of the diverter in the partially open fracture is proposed, as shown in Figure 15. Figure 15a shows that because of the good deformation performance, many fibers attach to the fracture surface and form a thin layer and extrude with the bridging particles to form a plugging zone. However, a large number of small particle diverters preferentially enter the fracture and fill the fracture tip, forming a dense plugging zone with the fibers and particles behind, as shown in Figure 15b. In addition, as shown in Figure 15c, if the fracture is long enough, the fibers will twine into masses and capture the following particles to form a loose plugging zone, and the flow channel narrows to plug completely, which is the most effective. It is worth noting that when the injected large particles can be bridged in front of the fracture tip and arranged in a row, forming a plugging zone with fibers and small particles, part of the empty area at the fracture tip is still unfilled, which weakens the plugging strength of the fracture tip, as shown in Figure 15d. It is worth noting that the combination of fibers and particles will better form bridges and fill gaps, thus increasing the plugging strength. It is necessary to analyze the specific application conditions of specific reservoirs to select the optimal combination formula of diverters for reservoirs with different pore and throat sizes.

### 3.6 Limitations and Prospect

This paper investigates the plugging performance and mechanism of the diverter commonly used in the field operation in partially open fractures, which can provide experimental and theoretical reference for increasing production in the field fracturing and make some progress. However, due to the limitations of experimental instruments, there are also some shortcomings, which can be investigated in the future.

(1) In the field operation, the length of the fracture is much longer than in the 3D printing model, and there will be many branch fractures that will change the flow trace and affect the concentration of the diverter, which can be further studied.
(2) The influence of complex injection schemes on plugging behavior can be considered in the future.
(3) According to the different types of reservoir lithology, there may be different plugging behaviors, which can be investigated in the future.

### 3.7 Conclusions

In this paper, based on the 3D printing method, partially open fractures are reproduced, the plugging law of diverter in fractures is investigated, and the plugging mechanism of partially open fractures is revealed. Through the analysis of the experimental results, the following conclusions are drawn:

(1) The surface morphology of partially open fractures is similar to that of open fractures, but the fracture width decreases sharply and the flow resistance increases significantly.
(2) Compared with a single fiber, the combination of fibers and particles facilitates the transport of diverter in the fracture to the tip and forms an effective plugging.
(3) When the particle size is half of the fracture width, the plugging effect is better. The optimal diverter formulas were different for partially open fractures with different fracture widths. The optimal diverter formulations for partially open fractures of 1, 2, and 4 mm were 1% F + 1% 0.15 mm P, 1% F + 1% 0.15 mm P + 1% 1 mm P, and 1% F + 1% 0.15 mm P + 1% 1 mm P + 1% 2 mm P.
(4) The plugging behavior of partially open fractures is different from that of open fractures, and the width of partially open fractures decreases sharply, which is beneficial to the bridging between particles and the adhesion between fibers, and the plugging is easier. The consumption of diverter for partially open fracture plugging is only one-third of that for an open fracture.
(5) The plugging mechanism of the diverter at the fracture tip is revealed, and four modes of classification are used to describe the plugging behavior of the diverter more comprehensively than before.
